# BDCD: a comprehensive Brain Disease Cell-cell communication Database

**DOI:** 10.1093/database/baag017

**Published:** 2026-04-02

**Authors:** Xinyi Liu, Citu Citu, Gang Qu, Wendao Liu, Nitesh Enduru, Andi Liu, Chia-Hao Tung, Zhongming Zhao

**Affiliations:** Center for Precision Health, McWilliams School of Biomedical Informatics, The University of Texas Health Science Center at Houston, Houston, TX 77030, United States; Center for Precision Health, McWilliams School of Biomedical Informatics, The University of Texas Health Science Center at Houston, Houston, TX 77030, United States; Center for Precision Health, McWilliams School of Biomedical Informatics, The University of Texas Health Science Center at Houston, Houston, TX 77030, United States; Center for Precision Health, McWilliams School of Biomedical Informatics, The University of Texas Health Science Center at Houston, Houston, TX 77030, United States; MD Anderson Cancer Center UTHealth Graduate School of Biomedical Sciences, Houston, TX 77030, United States; Center for Precision Health, McWilliams School of Biomedical Informatics, The University of Texas Health Science Center at Houston, Houston, TX 77030, United States; Center for Precision Health, McWilliams School of Biomedical Informatics, The University of Texas Health Science Center at Houston, Houston, TX 77030, United States; Stanford Knight Initiative for Brain Resilience, Stanford University, Stanford, CA 94305, United States; Center for Precision Health, McWilliams School of Biomedical Informatics, The University of Texas Health Science Center at Houston, Houston, TX 77030, United States; Center for Precision Health, McWilliams School of Biomedical Informatics, The University of Texas Health Science Center at Houston, Houston, TX 77030, United States; MD Anderson Cancer Center UTHealth Graduate School of Biomedical Sciences, Houston, TX 77030, United States

## Abstract

Dysregulated cell–cell communication (CCC) is increasingly recognized as a driver of brain disease pathology, contributing to neuroinflammation, synaptic dysfunction, and neurodegeneration. Nevertheless, existing resources remain limited in brain specificity, regional coverage, and functional annotation. To address this gap, we develop the Brain Disease Cell-cell communication Database (BDCD), the first comprehensive resource focused on CCC networks across major brain diseases. BDCD integrates 38 manually curated datasets, comprising 8 519 425 single cells from single-cell RNA-seq studies and 140 744 spots from spatial transcriptomic maps, spanning 14 brain regions and 13 canonical cell types covering Alzheimer’s disease, Parkinson’s disease, schizophrenia, bipolar disorder, and multiple sclerosis. BDCD reconstructs more than 495 000 ligand–receptor interaction events and links them to structural features, genetic associations, pathways, and therapeutic modulators, including 6100 single nucleotide polymorphisms from genome-wide association studies, 3350 drugs, and 72 477 allosteric modulators. This comprehensive atlas enables cross-disease comparison and supports dynamic hypothesis generation, providing a foundation for mechanistic insights and therapeutic discovery. BDCD is publicly available at https://bioinfo.uth.edu/bdcd/. **Database URL**: https://bioinfo.uth.edu/bdcd/

## Introduction

Brain diseases, such as Alzheimer’s disease (AD), Parkinson’s disease (PD), schizophrenia (SCZ), and bipolar disorder (BD), contribute significantly to the global health burden [1–3]. For instance, AD alone accounts for >36 million disability-adjusted life years (DALYs) worldwide, reflecting its profound societal and clinical impact [4]. These disorders disrupt brain-immune and neuroendocrine signalling pathways, leading to both cognitive and systemic impairments [5]. At the heart of these conditions lies the brain’s intricate cellular architecture, an expansive network of neurons, glial cells, and blood vessels connected through trillions of synapses [6, 7]. These diverse cell types constantly communicate via secreted ligands, membrane-bound receptors, and downstream signalling cascades to maintain brain homeostasis, plasticity, and immune surveillance [8]. Disruptions in such cell–cell communication (CCC) networks have now been recognized as central drivers of disease pathogenesis, not just downstream consequences [9, 10].

Increasing evidence indicates that CCCs, particularly ligand–receptor interactions (LRIs), are extensively rewired in diverse brain diseases, establishing aberrant signalling pathways that drive neuroinflammation, synaptic dysfunction, and neurodegeneration [11]. In AD, for instance, microglia transition into disease-associated microglia (DAMs) by rewiring the TREM2-APOE axis, profoundly affecting neuronal survival [12]. In PD, miswired astrocyte-neuron G protein-coupled receptor (GPCR) signalling via the adenosine A1 receptor (A1R)-α-synuclein axis contributes to α-synuclein aggregation, a process that may be targetable with A1R antagonists [13]. In neuropsychiatric conditions such as SCZ, CCC rewiring includes microglia-oligodendrocyte precursor cell (OPC) signalling via the TNF-TNFRSF1A axis to promote an inflammatory microenvironment that impairs myelination [14]. Disruption of NRG1-ERBB4 signalling between neurons and interneurons also impairs cortical circuits and gamma oscillations [15, 16]. Collectively, these findings demonstrate that CCC rewiring is not merely a secondary consequence of disease but a central mechanism driving pathogenic cellular behaviours and network-level dysregulation. Systematically outlining these altered communication pathways across brain regions and disease stages is thus critical for both mechanistic understanding and therapeutic innovation [17].

Rapid advancements in single-cell and spatial transcriptomic technologies have enabled high-resolution mapping of the brain’s cellular architecture and signalling networks [18, 19], providing crucial insights into dysregulated CCC in brain diseases [17]. Alongside these technologies, a growing number of computational tools, such as CellChat [20], CellPhoneDB [21], NicheNet [22], and LIANA+ [23], have been developed to systematically infer CCC events between cell types based on transcriptomic data. These CCCs represent signalling events transmitted between source and target cells through direct contact or secreted LRIs, enabling diverse brain cell types to coordinate processes such as synaptic plasticity, immune surveillance, and tissue maintenance [9, 24]. Reconstruction of CCC networks from transcriptomic data has thus become essential for uncovering how intercellular signalling governs brain development, maintains physiological balance, and responds to pathological perturbations [8].

Although CCCs are increasingly recognized as key regulators in brain pathology, existing resources remain inadequate for capturing their full complexity, due to several critical limitations:

(i) *Data fragmentation*. Existing CCC databases often lack brain-specific datasets (e.g. CellCommuNet) [25], while brain-focused resources typically centre on a single disease and treat intercellular signalling as a supplementary module (e.g. TACA [26]), hindering cross-disease or pan-brain comparisons.
(ii) *Lack of spatiotemporal resolution*. Current platforms fail to support comprehensive mapping of CCC networks across diverse brain regions or disease stages (e.g. Braak 0–VI), making it difficult to capture region-specific or stage-specific rewiring patterns.
(iii) *Limited functional annotation*. Dysregulated LRIs are rarely connected to broader biological features such as 3D protein structures, disease-associated variants [e.g. single nucleotide polymorphisms (SNPs) at the genome-wide significance level from genome-wide association studies (GWAS)], or drug/intervention libraries, constraining both mechanistic insights and translational utility.
(iv) *Absence of CCC plasticity modelling*. Most resources present static snapshots of CCCs, lacking tools to investigate how communication networks dynamically adapt to pathological stressors, environmental stimuli, or pharmacological interventions—factors critical to understanding disease progression and therapeutic response [27].

**Figure 1:**
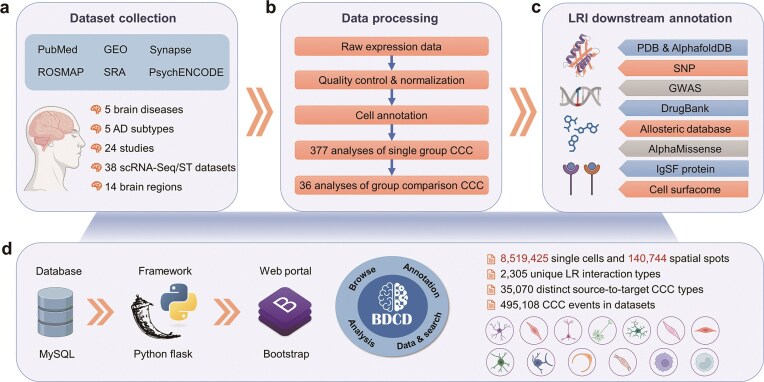
Overview of data collection, computational analysis, and downstream annotation of BDCD. AD: Alzheimer’s disease. ST: spatial transcriptomics. CCC: cell–cell communication. LRI: ligand–receptor interaction. GWAS: genome-wide association studies. LR: ligand receptor. SNP: single nucleotide polymorphism. IgSF: immunoglobulin superfamily protein.

To bridge these gaps, there is an urgent need for a brain-specific CCC resource that systematically captures communication dynamics across multiple regions, disease stages, and disorders, while also offering deeper functional annotations of LRIs. Here, we developed the Brain Disease Cell-cell communication Database (BDCD), the first database dedicated to CCC in brain diseases. BDCD integrates CCC networks reconstructed from 38 manually curated single-cell and spatial transcriptomic datasets derived from 24 landmark studies, encompassing five major brain diseases and 14 brain regions (Fig. 1). In BDCD, CCC events are computationally inferred from transcriptomic data and represent predicted signalling potential rather than experimentally validated or causal interactions. Therefore, BDCD is intended to support hypothesis generation and exploratory analysis, rather than definitive biological confirmation. Beyond CCC networks, BDCD provides multilevel annotations for each ligand and receptor, including their structural features, disease associations, druggability, and perturbation responsiveness, facilitating both mechanistic discovery and translational research. By offering a unified, annotated, and disease-aware atlas of brain CCC networks, BDCD aims to support dynamic hypothesis generation and guide future efforts in therapeutic targeting.

## Materials and methods

### Data collection and processing

An overview of dataset collection sources and summary statistics is shown in Fig. 1a. We included 24 single-cell/nucleus RNA sequencing (sc/snRNA-seq) and spatial transcriptomic studies from six major sources, including GEO [28], SYNAPSE [29], ROSMAP [30], SRA [31], and PsychENCODE [32], yielding 38 datasets organized by brain regions and disease types. Detailed metadata for each dataset, including accession numbers, disease types, brain regions, cell types, and sample sizes, are provided in Supplementary Table S1. The raw FASTQ files from sc/snRNA-seq were aligned to the GRCh38 human reference genome with GENCODE v48 [33] using Cell Ranger v7.1.0 [34] to generate gene expression matrices. When the FASTQ files were unavailable or datasets were prohibitively large to reprocess (e.g. Fujita *et al*. [35], Mathys *et al*. [36], and Mathys *et al*. [37]), we adopted the processed gene expression matrices and annotations provided by the original authors. Metadata consistency across studies was manually inspected and curated to ensure uniform annotation standards, enabling structured cross-dataset exploration.

### Quality control and normalization

To ensure data quality and consistency, we applied standard quality control and normalization procedures to the raw gene expression matrices. The filtering of low-quality cells and normalization of each sn/scRNA-seq dataset separately were performed by using Seurat 4.4.0 [38] in R, while Seurat 5.1.0 was used for spatial transcriptomics datasets (Fig. 1b). Specifically, cells with <200 or >2500 detected features, or with >5% mitochondrial gene content, were excluded. The filtered matrices were then log-normalized and scaled using Seurat’s *NormalizeData* and *ScaleData* functions. For large-scale datasets, we performed quality filtering and normalization using Scanpy 1.10.3 [39]. To maintain consistency across all datasets, the same thresholds were applied for cell filtering, and normalization using the *pp.normalize_total* and *pp.log1p* functions, which are functionally analogous to Seurat’s normalization workflow.

### Cell annotations

To ensure consistent and comparable cell-type labelling across datasets, we performed cell-type annotations using Seurat’s *FindTransferAnchors* and *MapQuery* functions, leveraging a well-characterized reference atlas from the SEA-AD consortium [40]. The reference includes eight canonical brain cell types: astrocytes, endothelial cells, excitatory neurons, inhibitory neurons, microglia, oligodendrocytes, OPCs, and vascular/leptomeningeal cells (VLMCs). Only cells with prediction scores ≥0.95 were retained for downstream analysis. For large-scale datasets, we adopted author-provided cell annotations, harmonized them with the reference-based categories described above. Additional relevant subtypes (e.g. macrophages, monocytes, and fibroblasts) were curated to enhance resolution in CCC analysis, while low-confidence or ambiguous labels (e.g. ‘unknown’) were excluded to ensure accuracy in downstream CCC analysis.

### Extraction of ligand–receptor interactions

For each sc/snRNA-seq dataset, we extracted 3234 human LRIs from CellChatDB 2.1.2 [20]. To reduce large dataset computational burden and ensure compatibility with downstream tools, we first subset the expression matrices processed via Scanpy using the gene symbol list of CellChatDB. The filtered matrices were saved in ‘.h5ad’ format and converted into ‘.rds’ format using the sceasy v0.0.7 package [41]. This standardized preprocessing allowed uniform analysis across all datasets using CellChat in R.

For spatial transcriptomic datasets, we used the LRI library provided by SpaTalk 1.0 [42], which includes 3398 LRI pairs. The use of pre-curated interaction libraries ensured consistency in downstream comparative analyses across different data modalities.

### Condition-specific grouping strategy

To enable condition-specific inference of CCC, we extracted relevant metadata for each dataset and defined multiple grouping strategies, including disease vs. control, sex, APOE ε4 status (present vs. absent), and APOE ε4 allele count (0, 1, or 2). For AD datasets that included Braak stage annotations, we defined three progressive categories: Braak 0/I/II as control, III/IV as intermediate, and V/VI as AD. These stratifications were used to perform both single-group and multiple-group comparison CCC analyses.

### CCC analysis

For each sc/snRNA-seq dataset, single-group CCC analysis was first performed using CellChat 2.1.2 with cell types as grouping labels. Within each condition (e.g. AD, control, male, or APOE ε4+), LRIs were identified based on default parameters. CCC events with probability ≥0.01 and *P* < .05 were retained and annotated with ligand, receptor, source/target cell types, and pathway. Next, to assess condition-specific differences, group comparison CCC analysis under predefined conditions (e.g. disease vs. control) was performed by merging CellChat objects using the *mergeCellChat* function. LRIs and signalling pathways with adjusted *P*-value < .05 were considered statistically significant, thereby indicating rewired CCC events that capture condition-dependent changes in intercellular communication patterns within each dataset. Briefly, pathway communication probability represents the aggregated communication probability of all LRIs belonging to a given signalling pathway, as computed by CellChat. Specifically, CellChat first estimates communication probabilities for individual LR pairs based on expression and permutation testing, and these values are next aggregated to derive pathway-level communication strength (i.e. pathway ‘information flow’), summarizing LR communication probabilities within each signalling pathway.

For spatial transcriptomics analysis, we analysed five spot-based datasets generated using the 10x Visium platform. Pre-annotated Seurat objects containing spot-level cell-type information were used as input. SpaTalk 1.0 was chosen for its compatibility with spot-based data. Each brain tissue sample was analysed independently to preserve spatial resolution and context. LRIs were extracted using SpaTalk’s default parameters and built-in library. The spatial CCC inferred from 10x Visium data represents spot-level communication potential, rather than interactions between individual cells, as each spot may contain multiple cell types. Accordingly, the spatial resolution of CCC inference is inherently constrained by the Visium platform. In this context, CCC rewiring in spatial transcriptomic datasets is characterized at the sample level, reflecting differences in inferred CCC events across individuals or conditions, rather than formal group-level statistical comparison.

CCC analyses were conducted independently within each dataset. Communication probabilities were not directly compared across datasets unless derived from the same study and experimental context, and cross-dataset exploration is intended for qualitative pattern discovery rather than direct quantitative comparison. Overall, CCC events in BDCD were computationally inferred from transcriptomic LR expression patterns using CellChat and SpaTalk and should be interpreted as predicted signalling potential, not experimentally validated interactions.

### Ligand/receptor annotation and interpretation

We curated 2305 LRIs from CellChatDB and SpaTalk analysis and annotated ligand/receptor proteins with UniProt biological functions [43], PDB [44] and AlphaFoldDB structures [45], cell surface, and immunoglobulin superfamily (IgSF) protein information [46]. Genetic insights were integrated using the SNPs at the genome-wide significance level (*P* < 5 × 10⁻⁸) from the NHGRI-EBI GWAS Catalog [47]. Trait annotations were manually curated to retain brain-related, neurological, psychiatric, and metabolism-associated phenotypes relevant to brain disease mechanisms, while excluding broad anthropometric or non-specific traits. SNPs were linked to ligand and receptor genes based on reported mapped genes (harmonized to HGNC symbols) and intersected with curated ligand/receptor gene lists. This annotation reflects locus-level genetic relevance and does not imply causality or fine-mapped targets [48]. In addition, AlphaMissense pathogenicity scores [49] were incorporated as computational predictions of missense variant pathogenicity, providing an additional layer of functional interpretation for ligand and receptor genes. Therapeutic relevance was annotated using drug–target relationships from DrugBank 5.1.12 [50], and allosteric modulators from ASD 5.0 [51], supporting target prioritization (Fig. 1c).

### Database construction and web portal interface

The BDCD web portal was built using JavaScript, Python, and MySQL (Fig. 1d). Bootstrap 5.3.0 was used to organize the webpage components. jQuery 3.6.0 was utilized for query backend, event handling, and DOM manipulation. Echarts 5.4.0, htmlwidgets 1.6.4, and plotly.js 2.21.0 were employed to generate and display charts; Flask 3.0.3 in Python 3.9.20 was used to build the backend of the BDCD, and all datasets were stored in MySQL 5.5.56.

### Data statistics

BDCD provides structured intercellular signalling data from 8 519 425 human brain cells obtained by sc/sn RNA-seq and 140 744 spots from spatial transcriptomic (ST) datasets, compiled from total 38 curated datasets from 24 studies, including 34 sc/sn RNA-seq and 4 ST datasets. As summarized in Table 1, these datasets span five major brain diseases: AD, PD, MS, SCZ, and BD, covering 14 brain regions and 13 canonical cell types. For AD datasets, BDCD includes comprehensive coverage across disease subtypes and stages, including late-onset Alzheimer’s disease (LOAD), early-onset Alzheimer’s disease (EOAD), familial Alzheimer’s disease (fAD), sporadic Alzheimer’s disease (sAD) and Down syndrome Alzheimer’s disease (DSAD) cases, and related biological conditions including Braak stage, sex, APOE ε4 genotype, and disease status. In total, we performed 413 individual CCC analyses across datasets and predefined condition groupings.

**Table 1:** Statistical summary of BDCD database.

Data type	Counts
Single cells/nuclei	8 519 425
Spatial transcriptomic spots	140 744
Brain disease datasets	38
Brain regions	14
Cell types	13
Ligand–receptor interaction types	2 305
Cell–cell communication types	35 070
Cell–cell communication events	495 108
Pathways for cell–cell communication	109
Ligand/receptor proteins	941
SNP and GWAS records	6 100
Potential drugs	3 350
Allosteric modulators	72 477

All BDCD records are publicly available at https://bioinfo.uth.edu/bdcd/. Users can download tabular results in TXT or CSV format, including the following records:

1. *Dataset metadata (38 entries)*. Includes disease, region, sample size, cell counts, sequencing modality, and publication details.
2. *Analysis condition metadata (413 entries)*. Each dataset is annotated with one or more condition groups (e.g. AD vs. control, male vs. female), with grouping variables and case/control definitions.
3. *Cell-type-level CCC events (495 108 entries)*. For each condition or comparison, significant ligand–receptor interactions are recorded with source/target cell types, interaction probability, *P*-value, and pathway membership.
4. *Ligand–receptor interaction annotation (2305 LRIs)*. Each LRI is linked to UniProt IDs, 3D structures (PDB/AlphaFoldDB), AlphaMissense scores, and therapeutic annotations from DrugBank (3350 drugs, 72 477 allosteric modulators).
5. *Protein annotation tables*. Ligands and receptors including cell-surface marker tags and domain family classifications (e.g. IgSF).

### Web interface and functionality

**Figure 2:**
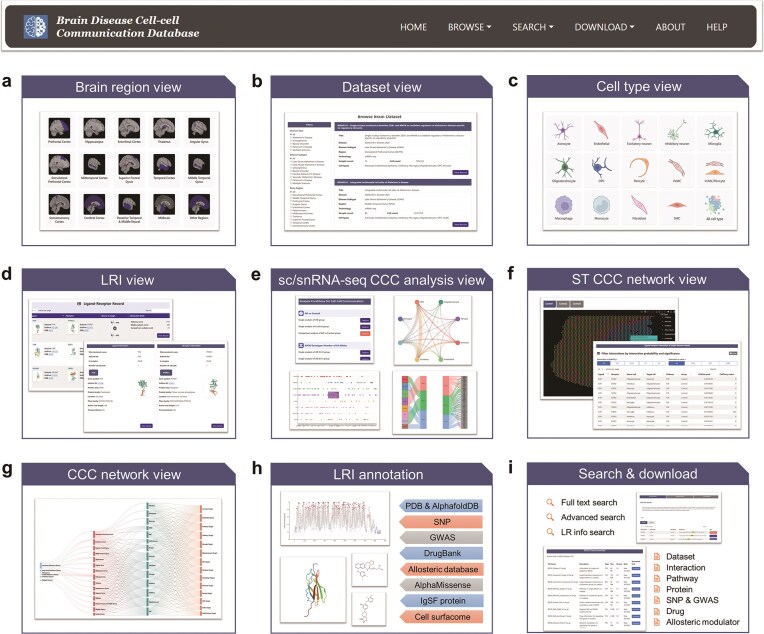
Overview of BDCD web interface. BDCD portal offers an intuitive interface to explore brain disease-related cell–cell communication (CCC) data. Users can browse CCC data by brain region (a), dataset (b), or cell type (c). The platform offers interactive views of ligand–receptor interactions (LRI) (d), as well as condition-specific CCC analyses derived from single-cell/nucleus RNA sequencing (sc/snRNA-seq) (e) and spatial transcriptomics (ST) datasets (f). The network view (g) presents a comprehensive visualization of intercellular communication. Additionally, LRI annotation modules (h) integrate structural, genomic, and pharmacological information from databases such as PDB, GWAS, DrugBank, and others. Multiple search and download options (i), including full-text, advanced, and LRI-specific queries, support flexible data access and retrieval.

BDCD provides a detailed step-by-step tutorial at https://bioinfo.uth.edu/bdcd/help. Through a user-friendly web interface (Fig. 2), users can access all processed CCC data with four browse modes, as described below:

1. *Dataset view*. This panel enables users to explore each curated brain disease dataset through comprehensive metadata, including disease type, brain region, sample size, and analysis conditions (Fig. 2a and b). For each sc/snRNA-seq and ST dataset, results from both single-group and comparative CCC analyses are systematically integrated (Fig. 2e and f). Users can interactively examine CCC networks, signalling pathways, and LRIs across conditions such as disease vs. control, sex, APOE ε4 status, and Braak stage, aided by interactive plots and filterable tables.
2. *Cell view*. This panel summarizes CCC activity across 13 brain cell types and links to related datasets and LRIs (Fig. 2c).
3. *Interaction view*. This view enables gene and cell-type specific exploration of LRI records (Fig. 2d). For each LRI, BDCD provides a comprehensive list of communication events across source–target cell pair contexts. Users can compare the rewiring of the same LRI across multiple datasets and conditions. Each LRI is also linked to downstream annotations including 3D structure, SNPs, mutations, drugs, allosteric modulators, and cell surface localization.
4. *Network view*. This option visualizes directional CCC networks across diseases, brain regions, and interacting cell types (Fig. 2g).

Additionally, all ligands and receptors included in BDCD are annotated with comprehensive information, as illustrated in Fig. 2h. These annotations cover protein structures (PDB and AlphaFoldDB), genetic variants (SNP, GWAS, and AlphaMissense), drug associations (DrugBank and allosteric modulators), IgSF protein, and cell surfaceome information. This enables users to conduct in-depth exploration of the functional and structural characteristics of each gene or protein.

The ‘Search’ module offers flexible access through three modes: ‘Full text search’ for general queries, ‘Advanced search’ with filters for disease, region, or cell type, and LR info search for targeting specific LRI. The ‘Download’ module provides all core datasets and annotations, including interactions, pathways, proteins, SNPs, drugs, and modulators, in TXT/CSV format for offline use (Fig. 2i).

The BDCD portal was tested for usability, accessibility, and security. The system functions reliably across major browsers (Chrome, Edge, Safari, and Firefox) and on both desktop and mobile devices. Accessibility was confirmed via Siteimprove and keyboard navigation, meeting WCAG 2.1 Level AA. Security checks ensured HTTPS, protected downloads, and no major vulnerabilities. These validations support a reliable, safe, and user-friendly experience.

### Data validation

To validate the accuracy, utility, and reproducibility of BDCD, we conducted a series of assessments on the curated datasets. As an initial step, we evaluated the cell-type composition across all 38 single-cell and spatial transcriptomic datasets. For consistent comparisons, eight major canonical brain cell types were selected for unified analysis: astrocytes (Ast), oligodendrocytes (Oli), OPCs (Opc), excitatory neurons (Exc), inhibitory neurons (Inh), microglia (Mic), endothelial cells (End), and pericytes (Per).

**Figure 3:**
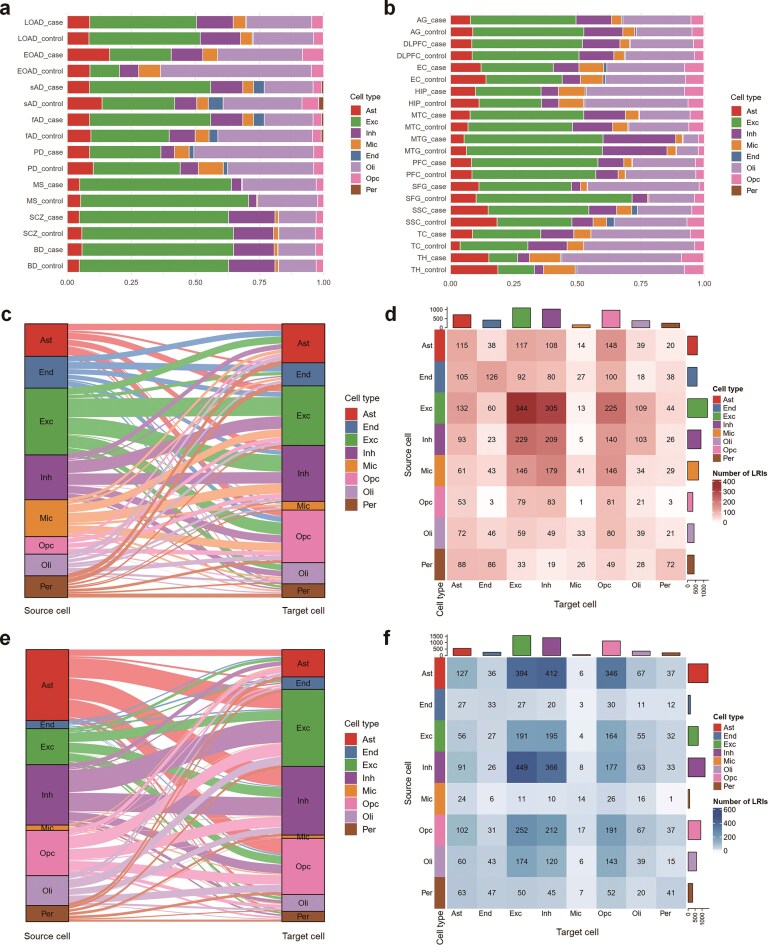
Analysis of cell-type composition and rewired cell–cell communication (CCC) ligand–receptor interaction (LRI) pairs between eight major brain cell types in comparative analysis from BDCD. (a) Cell type proportions of sc/snRNA-seq datasets across eight disease conditions, including four subtypes of Alzheimer’s disease (AD): late-onset Alzheimer’s disease (LOAD), early-onset Alzheimer’s disease (EOAD), sporadic Alzheimer’s disease (sAD), and familial Alzheimer’s disease (fAD), as well as Parkinson’s disease (PD), multiple sclerosis (MS), schizophrenia (SCZ), and bipolar disorder (BD). (b) Cell-type composition across anatomically and functionally defined brain regions. Brain regions included in this study were the angular gyrus (AG), dorsolateral prefrontal cortex (DLPFC), entorhinal cortex (EC), hippocampus (HIP), middle temporal cortex (MTC), middle temporal gyrus (MTG), prefrontal cortex (PFC), superior frontal gyrus (SFG), somatosensory cortex (SSC), temporal cortex (TC), and thalamus (TH). (c) Rewired CCC Sankey plot for significantly upregulated LRIs between source and target cell types. (d) Heatmap showing the eight number of upregulated LRIs. (e) CCC Sankey plot for downregulated LRIs. (f) Heatmap for downregulated LRIs.

We then compared the distribution of these cell types across eight brain disorders and their respective control groups, including four AD subtypes, PD, MS, SCZ, and BD (Fig. 3a). Notably, we observed a pronounced reduction in the proportion of excitatory neurons in the case groups of LOAD and PD, consistent with well-established reports of neurodegeneration in these conditions [52]. In EOAD cases, both astrocytes and microglia showed increased proportions, suggesting heightened glial reactivity and inflammation, consistent with gliosis and neuroinflammatory responses reported in AD pathology [53]. We further investigated cell-type composition across different anatomical regions (Fig. 3b). In the prefrontal cortex (PFC), oligodendrocyte proportions were noticeably reduced in disease samples, consistent with previous reports of myelin degeneration and oligodendrocyte dysfunction in both AD and neuropsychiatric disorders [54,55]. These alterations may contribute to disrupted axonal conduction and cognitive decline [56]. In the superior frontal gyrus (SFG), excitatory neuron proportions markedly decreased in disease samples, consistent with reports of neuronal loss and cortical atrophy in disease progression [57].

To systematically summarize the extent of CCC alterations across brain diseases, we focused on datasets suitable for comparative CCC analysis and performed condition-specific differential expression independently within each dataset. This design minimizes potential biases arising from heterogeneity in data quality and cell-type composition. We extracted a filtered CCC event subset of BDCD with a merged probability ≥0.01, adjusted *P*-value < .05, and absolute ligand log_2_FC > 0.15 across comparison groups. We next summarized the number of non-redundant, significantly upregulated, or downregulated LRIs across all datasets. To visualize the directional patterns of these interactions, we generated aggregate CCC Sankey plots and corresponding heatmaps that reflect the frequency and intensity of communication between cell-type pairs (Fig. 3c–f). The edge width in the Sankey plots corresponds to the number of significant LRIs between source and target cell types. The upregulated CCC plot (Fig. 3c and d) shows that excitatory and inhibitory neurons exhibited the highest number of outgoing signals, particularly towards each other and astrocytes, indicating enhanced neuronal-glial feedback under disease conditions [58].

Astrocytes have increasingly been recognized as a key cellular target in brain diseases, receiving a large number of upregulated LRIs from neurons and OPCs, consistent with their known roles in synaptic and metabolic regulation during neuroinflammation and degeneration [59]. In parallel, the downregulated CCC Sankey plot (Fig. 3e and f) indicated a distinct subset of suppressed interactions. Notably, many neuron-to-glia interactions were attenuated, especially from excitatory neurons to astrocytes and endothelial cells. Astrocytes were also broadly affected as both senders and receivers, reflecting a dampened communication profile potentially associated with functional loss in chronic disease states [60]. Furthermore, microglia-to-multiple-cell-type interactions were frequently downregulated, suggesting possible transitions towards immune resolution or dysfunction [61]. These two directional layers together underscore complex rewiring patterns that selectively enhance or suppress CCC among specific cell-type pairs during disease.

**Figure 4:**
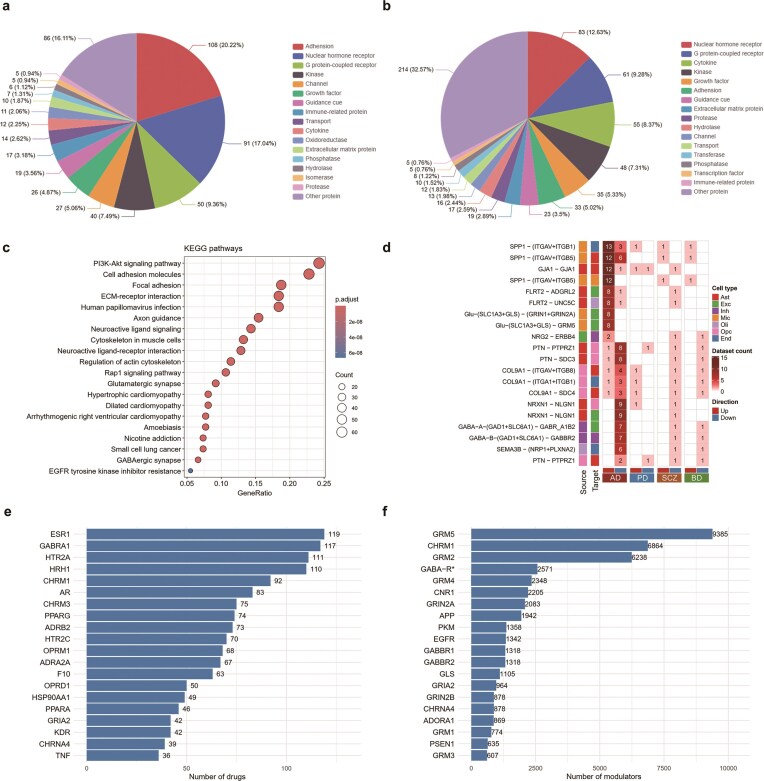
Functional characterization and validation of dysregulated LRIs. (a) Distribution of protein class of ligand/receptor genes in sc/snRNA-seq datasets. (b) Distribution of protein class of ligand/receptor genes in spatial transcriptomics datasets. (c) KEGG pathway enrichment of rewired ligand/receptor genes identified from group comparison cell–cell communication analysis of sc/snRNA-seq. (d) Representative LRIs frequently dysregulated across diseases, with matrix colour indicating direction (red: upregulated; blue: downregulatedand count indicating dataset frequency. (e, f) Barplots for top LRI components ranked by the number of known drugs (e) and allosteric modulators (f).

To further characterize the molecular properties of disease-relevant CCC events, we analysed the protein class distribution of LRIs separately for sc/snRNA-seq (Fig. 4a) and ST datasets (Fig. 4b). Because these two modalities were processed using distinct tools and algorithms, and the relative fraction of proteins categorized as ‘Other protein’ varied between datasets, we evaluated them independently to ensure methodological consistency. Notably, both analyses revealed highly concordant trends: adhesion molecules (scRNA-seq: 20.2%; ST: 12.6%), nuclear hormone receptors (scRNA-seq: 17.0%; ST: 25.7%), and GPCRs (scRNA-seq: 9.4%; ST: 8.3%) were consistently among the most enriched categories. These results highlight their central roles in mediating CCC, transcriptional regulation, and signal transduction during disease, and further confirm the robustness of our findings across different technologies. We further performed KEGG enrichment analysis using the genes underlying rewired CCC events identified from our sc/snRNA-seq group comparison (Fig. 4c). This analysis revealed significant overrepresentation of pathways involved in neuroinflammation, synaptic transmission, and immune modulation, such as the PI3K-Akt signalling pathway [62], ECM–receptor interaction [63], and glutamatergic/GABAergic synapses [64, 65].

We further summarized the most frequently dysregulated LRIs across datasets and disease types, highlighting conserved communication patterns that may represent key regulatory hubs in brain disorders (Fig. 4d). For instance, SPP1-ITGAV+ITGB1, a microglia-derived integrin ligand pair, was frequently upregulated and is known to promote neuroinflammation and phagocytosis in AD and PD [66]. The glutamatergic axis Glu-(SLC1A3+GLS)-(GRIN1+GRIN2A)/GRM5 reflects excitatory signalling between astrocytes and neurons; both GRIN2A and GRM5 are well-established drug targets in neurodegeneration and psychiatric disorders (Fig. 4d and f) [67, 68]. Dysregulated GABAergic pairs such as GABA-A-(GAD1+SLC6A1)-GABRA1B2 or GABBR2 highlight impaired inhibitory signalling in AD and SCZ [65, 69]. Additionally, the FLRT2-ADGRL2 interaction, an adhesion GPCR pair, was consistently upregulated across conditions and may represent an emerging therapeutic target involved in neuronal circuit remodelling [70].

## Conclusion

In this study, we developed BDCD, a comprehensive and user-friendly database dedicated to investigating CCC alterations associated with diverse brain diseases. BDCD integrates 38 manually curated datasets and systematically standardizes metadata, cell-type annotations, and communication signals, enabling direct and biologically consistent comparisons across conditions within the same dataset or study context. The web platform provides intuitive visualizations, interactive query modules, and rich displays of analytical results, allowing users to explore LRIs, cell-type specificity, and CCC patterns at multiple resolutions. Through data validation and utility assessment, we demonstrate that BDCD preserves brain disease-associated communication signatures, including cell composition trends, dominant signalling ligands and pathways, and multicellular coordination modules. These results support the reliability of BDCD as a resource for investigating both conserved and disease-specific CCC patterns.

Specifically, CCC analyses in BDCD are designed to be interpreted within the context of individual datasets or within-study comparisons. Cross-dataset exploration is intended to facilitate qualitative pattern discovery and hypothesis generation, rather than direct quantitative comparison of communication strengths across independent studies.

By providing standardized data processing, transparent analytical logic, and application-oriented visualization, BDCD serves as a practical tool for hypothesis generation, mechanistic exploration, and translational research. To ensure that BDCD remains at the forefront of brain disease CCC research, the database will be updated every 6 months, with interim updates implemented as major new brain single-cell or spatial transcriptomic datasets become available. Each release will include versioning and brief release notes. Furthermore, we plan to integrate a wider array of CCC inference algorithms and multi-omics data layers. This continuous enhancement will empower researchers to uncover novel pathological mechanisms and identify promising therapeutic targets from an expandable, multimodal atlas of brain cell–cell communication.

## Author contributions

Conceptualization: Z.Z.; methodology: X.L., C.C., G.Q., W.L., A.L., and Z.Z.; formal analysis: X.L., C.C., G.Q., W.L., N.E., A.L., and C.H.-T.; investigation: Z.Z; resources: Z.Z; data curation: X.L., C.C., N.E., and A.L.; writing—original draft: X.L.; writing—review & editing: X.L., C.C., G.Q., W.L., and Z.Z.; visualization: X.L. and G.Q.; supervision: Z.Z.; project administration: Z.Z.; and funding acquisition: Z.Z.

## Supplementary Material

baag017_Supplemental_File

## Data Availability

All data described in this manuscript are available at https://bioinfo.uth.edu/bdcd/
